# DHA Suppresses Hepatic Lipid Accumulation *via Cyclin D1* in Zebrafish

**DOI:** 10.3389/fnut.2021.797510

**Published:** 2022-01-25

**Authors:** Qianwen Ding, Qiang Hao, Qingshuang Zhang, Yalin Yang, Rolf Erik Olsen, Einar Ringø, Chao Ran, Zhen Zhang, Zhigang Zhou

**Affiliations:** ^1^China-Norway Joint Lab on Fish Gastrointestinal Microbiota, Institute of Feed Research, Chinese Academy of Agricultural Sciences, Beijing, China; ^2^Norway-China Joint Lab on Fish Gastrointestinal Microbiota, Institute of Biology, Norwegian University of Science and Technology, Trondheim, Norway; ^3^Key Laboratory for Feed Biotechnology of the Ministry of Agriculture and Rural Affairs, Institute of Feed Research, Chinese Academy of Agricultural Sciences, Beijing, China

**Keywords:** high-fat diet, DHA, lipid accumulation, Cyclin D1, gut microbiota

## Abstract

With the widespread use of high-fat diets (HFDs) in aquaculture, fatty livers are frequently observed in many fish species. The aim of this study was to investigate if docosahexaenoic acid (DHA) could be used to reduce the fatty liver in zebrafish generated by a 16% soybean oil-HFD over 2 weeks of feeding. The DHA was added to iso-lipidic HFD at 0.5, 1.0, and 2.0% of diet. Supplementation of DHA reduced growth and feed efficiency in a dose dependent manner being lowest in the HFDHA2.0 group. Hepatic triglyceride (TG) in zebrafish fed 0.5% DHA-supplemented HFD (HFDHA0.5) was significantly lower than in the HFD control. Transcriptional analyses of hepatic genes showed that lipid synthesis was reduced, while fatty acid β-oxidation was increased in the HFDHA0.5 group. Furthermore, the expression of *Cyclin D1* in liver of zebrafish fed HFDHA0.5 was significantly reduced compared to that in fish fed HFD. In zebrafish liver cells, *Cyclin D1* knockdown and blocking of Cyclin D1-CDK4 signal led to inhibited lipid biosynthesis and elevated lipid β-oxidation. Besides, DHA-supplemented diet resulted in a rich of *Proteobacteria* and *Actinobacteriota* in gut microbiota, which promoted lipid β-oxidation but did not alter the expression of *Cyclin D1* in germ-free zebrafish model. In conclusion, DHA not only inhibits hepatic lipid synthesis and promotes lipid β-oxidation *via* Cyclin D1 inhibition, but also facilitates lipid β-oxidation *via* gut microbiota. This study reveals the lipid-lowering effects of DHA and highlights the importance of fatty acid composition when formulating fish HFD.

## Introduction

Feeding high-fat diets (HFDs) is common in many farmed fish species as it increases growth rate and feed conversion. However, HFDs are also known to generate fatty livers in many fish (1–6). Although it is unclear to what extent the fatty acid compositions of the oils affect the severity of fatty livers, it has been demonstrated that oils rich in saturated (SFA) or monounsaturated fatty acids (MUFAs) are more obesogenic than oils containing highly unsaturated *n* – 3 fatty acids (HUFAs) (7, 8). It appears that docosahexaenoic acid, 22:6*n* – 3 (DHA) in particular has a distinct anti-obesity effect as shown in both mice and zebrafish (7, 9). If this is correct, then the extensive replacement of fish oil in farmed fish diets by soybean oil, palm oil and coconut oil that are deficient in HUFAs (10–13) may predispose for a nutritional condition favoring the development of fatty livers.

In mammals, dietary interventions with DHA reduce the severity of non-alcoholic fatty liver disease (NAFLD) and acute ethanol-induced hepatic steatosis. This is linked to reduced lipid synthesis and increased fatty acid oxidation (14–18). The mechanism appears, in part, to be due to modulations of transcription factors including *Ppar*α, *Nrf2, Srebp-1c*, and *Nf*κ*b* (19, 20). DHA has therefore been suggested as a potential treatment for fatty liver disease (21). Current data on the potential anti-obesogenic effects of DHA in fish has focused on adipose tissues. The DHA requirement of warm water fish is about 0.5-2.6% of the diet (22). Dietary supplementation of 0.6% DHA reduces lipid accumulation *via* induction of adipocyte apoptosis (23). Using primary cultures of preadipocytes from cobia showed that DHA hinders preadipocyte differentiation and lipid accumulation from cobia (24). There is however a general lack of understanding of the underlying mechanisms on how DHA regulates hepatic lipid metabolism.

The main fate of dietary DHA in cells is to incorporate into membrane phospholipids (25–27). DHA can regulate basic membrane properties such as fluidity, permeability and protein activity (26), thus affecting cell growth and proliferation (28). The first gap (G1) phase is a period between nuclear division and DNA synthesis, during which various signals derived from metabolism, stress and environmental factors are integrated to determine the developmental program including cell division, differentiation or death (29). Cyclin D1, a critical target of proliferative signals in the G1 phase, plays an important role in the transition at end of G1 phase when cells move into the synthesis (S) phase just before cell division (30). In addition to its role in cell cycle progression, the high expression of Cyclin D1 in NAFLD and livers with microsteatosis suggests its possible importance in fatty liver diseases and lipid metabolism (31, 32). Moreover, the drug, metformin, used for treating NAFLD leads to inhibition of Cyclin D1 (33). A recent study in blunt snount bream (*Megalobrama amblycephala*) showed that dietary DHA induces liver cell cycle arrest at G1 phase (34). However, whether similar links exist between Cyclin D1 and lipid metabolism in fish is still uncertain.

In this study, we evaluated the effects of DHA on growth and hepatic lipid accumulation in zebrafish fed with three levels of DHA (0.5, 1.0, and 2.0%) in diets. We also measured the expression levels of genes related to lipid synthesis, fatty acid oxidation in the liver. The effects of DHA on the basic cell life processes were evaluated in zebrafish liver (ZFL) cells, which were further validated by analyzing the expression of *Cyclin*s. The roles of Cyclin D1 were also validated in a gene-knockdown ZFL model. Finally, the effect of gut microbiota and possible consequences of microbial changes on fish physiology were further assessed in HFD and 0.5% DHA-supplemented HFD (HFDHA0.5) groups by transferring gut micobiota to a germ-free (GF) zebrafish model.

## Materials and Methods

### Fish Husbandry

Zebrafish of the Tübingen strain were maintained at the zebrafish facility of the Institute of Feed Research, Chinese Academy of Agricultural Sciences (Beijing, China). The size of each tank was 25.5 × 18.5 × 18.0 cm. During the 2-wk feeding period, the water in the rearing system was kept running, the rearing temperature was 25-28°C, the dissolved oxygen was >6.0 mg/L, the pH was 7.0-7.2, the nitrogen content was <0.50 mg/L and the nitrogen content (as NO_2_) was <0.02 mg/L. Zebrafish were maintained at a 14:10 L:D cycle.

### Diets and Feeding Trial

Feed formulation and fatty acid composition of diets are presented in Tables 1, 2. Casein, soybean oil and wheat flour were used as dietary protein, lipid and carbohydrate sources, respectively. The low-fat diet (LFD) was supplemented with soybean oil with 60 g/kg and the HFD was supplemented with soybean oil with 160 g/kg (Table 1). The DHA used in the feeding trial was at purity of 90% (Larodan, 10-2206-90-13). Increased DHA levels were compensated by decreasing equal levels of soybean oil. All dry ingredients were ground through a 60-mesh screen. The diets were prepared by mixing the dry ingredients with the oil and water manually. Then each diet was extruded in a manual extruder with a 2.5-mm aperture. The extruded pellets were freeze-dried and stored at −20°C in plastic bags in small quantities. Before feeding, the feed pellets were ground through a 30-mesh screen.

**Table 1:** Ingredients of experimental diets for 1-month-old zebrafish (g/kg).

	**1-month-old zebrafish**				
**Ingredients (g/kg dry diet)**	**LFD**	**HFD**	**HFDHA0.5**	**HFDHA1.0**	**HFDHA2.0**
Casein	400	400	400	400	400
Geltin	100	100	100	100	100
Wheat flour	350	250	250	250	250
DHA^a^	0	0	5	10	20
Soybean oil	60	160	155	150	140
Lysine	3.3	3.3	3.3	3.3	3.3
Ascorbyl phosphater	1	1	1	1	1
Vitamin premix^b^	2	2	2	2	2
Mineral premix^c^	2	2	2	2	2
Monocalcium phosphate	20	20	20	20	20
Choline chloride	2	2	2	2	2
Sodium alginate	20	20	20	20	20
Microcrystalline cellulose	39.7	39.7	39.7	39.7	39.7
Total	1,000	1,000	1,000	1,000	1,000
**Proximate composition (g/kg dry diet)**					
Crude protein	458.9	459.3	463.6	459.5	463.6
Crude lipid	57.3	152.3	153.1	150.4	152.0
Ash	31.1	31.2	31.7	31.5	32

a *Larodan*.

b *Vitamin premix (g/kg): thiamine, 0.438; riboflavin, 0.632; pyridoxine·HCl, 0.908; d-pantothenic acid, 1.724; nicotinic acid, 4.583; biotin, 0.211; folic acid, 0.549; vitamin B-12, 0.001; inositol, 21.053; menadione sodium bisulfite, 0.889; retinyl acetate, 0.677; cholecalciferol, 0.116; dl-α-tocopherol-acetate, 12.632*.

c *Mineral premix (g/kg): CoCl_2_·6H_2_O, 0.074; CuSO_4_·5H_2_O, 2.5; FeSO_4_·7H_2_O, 73.2; NaCl, 40.0; MgSO_4_·7H_2_O, 284.0; MnSO_4_· H_2_O, 6.50; KI, 0.68; Na_2_SeO_3_, 0.10; ZnSO_4_·7H_2_O, 131.93; Cellulose, 501.09*.

*LFD, low-fat diet; HFD, high-fat diet; HFDHA, DHA-supplemented HFD*.

**Table 2:** Fatty acid compositions of the five diets in 1-month-old zebrafish (g/kg).

**Fatty acid, g/kg diet**	**LFD**	**HFD**	**HFDHA0.5**	**HFDHA1.0**	**HFDHA2.0**
C16:0	6.83 ± 0.79^c^	15.63 ± 0.06^ab^	17.09 ± 1.63^a^	16.55 ± 0.03^ab^	14.61 ± 0.05^b^
C18:0	3.00 ± 0.35^b^	6.82 ± 0.03^a^	6.46 ± 0.75^a^	7.21 ± 0.01^a^	6.43 ± 0.03^a^
Total saturates^1^	11.10 ± 1.28^c^	24.94 ± 0.05^ab^	26.02 ± 0.61^a^	26.51 ± 0.04^a^	23.54 ± 0.08^b^
C18:1	12.81 ± 1.69^b^	33.29 ± 0.14^a^	31.80 ± 3.96^a^	35.96 ± 0.08^a^	31.44 ± 0.16^a^
Total monoenes^2^	13.07 ± 1.71^b^	33.83 ± 0.14^a^	32.32 ± 4.01^a^	36.54 ± 0.08^a^	31.97 ± 0.17^a^
C18:2	25.64 ± 3.38^b^	67.85 ± 0.1^a^	64.99 ± 8.2^a^	73.50 ± 0.22^a^	64.14 ± 0.39^a^
Total (*n* – 6)^3^	25.67 ± 3.38^b^	67.91 ± 0.1^a^	65.04 ± 8.2^a^	73.56 ± 0.22^a^	64.19 ± 0.39^a^
C18:3	2.74 ± 0.27^b^	7.50 ± 0.02^a^	7.36 ± 0.9^a^	8.24 ± 0.03^a^	7.22 ± 0.01^a^
C22:6	0.00 ± 0.00^d^	0.00 ± 0.00^d^	3.40 ± 0.05^c^	5.14 ± 0.01^b^	14.31 ± 0.09^a^
Total (*n* – 3)^4^	2.74 ± 0.27^e^	7.50 ± 0.02^d^	10.82 ± 0.95^c^	13.46 ± 0.04^b^	21.73 ± 0.12^a^
Total PUFA	28.41 ± 3.65^b^	75.41 ± 0.08^a^	75.86 ± 9.15^a^	87.02 ± 0.26^a^	85.92 ± 0.52^a^
(*n* – 3):(*n* – 6)	0.11 ± 0.00^d^	0.11 ± 0.00^d^	0.17 ± 0.00^c^	0.18 ± 0.00^b^	0.34 ± 0.00^a^

*Values are means ± SEMs; n = 3. Means without a common letter are significantly different, P < 0.05*.

1 *includes 6:0, 8:0, 10:0, 12:0, 14:0, 15:0, 16:0, 17:0, 18:0, 20:0, 21:0, 22:0, 23:0, 24:0*.

2 *includes 14:1, 16:1, 20:1, 18:1, 22:1*.

3 *includes 18:2, 20:2*.

4 *includes 18:3, 20:5, 22:6*.

*LFD, low-fat diet; HFD, high-fat diet; HFDHA, DHA-supplemented HFD*.

During the feeding trial, healthy, uniformly sized 1-month-old zebrafish (1.160 ± 0.005 g/20 fish) were divided into five groups at random and fed the LFD, HFD, or 0.5, 1.0, 2.0% DHA-supplemented HFDs (HFDHA0.5, HFDHA1.0, HFDHA2.0, respectively; Table 1). The feed amount was at 6% of body weight, twice a day at 9:00 and 16:00. Each group contained three tanks with 20 fish per tank.

### Sample Collection and Analysis

All fish were anesthetized with tricaine methanesulfonate (MS222) before sampling. At the end of the 2-wk feeding trial, the survival rate in each group was recorded (100 × final survival individuals/initial individuals); the fish in each tank were weighed to calculate weight gain (100 × [final body weight – initial body weight]/initial body weight) and feed efficiency (100 × [final body weight – initial body weight]/feed intake). The liver was collected at 24 h after the last feeding for analysis of histology (H&E), triacylglycerol (TG) content, fatty acid compositions detection and gene expression. The gut contents were collected at the 4-6 h after the last feeding. The gut contents were collected under aseptic conditions. The digesta samples from the six fish were pooled as a replicate. The muscle was collected at 24 h after the last feeding for analysis of TG content and gene expression.

### Histological Analysis

The livers of zebrafish were rinsed with sterilized PBS, fixed in 4% formalin solution, and embedded in paraffin. For histological analysis, the embedded livers were cut into 5-8 μm sections and placed on slides. After dewaxing, hematoxylin and eosin were used to stain nucleus and cytoplasm, respectively. Following dehydration by alcohol and being transparent by xylene. For oil red staining, the embedded livers were cut into 10 μm sections and placed on slides. Liver sections were immersed by 60% isopropanol for 2 min. Then liver sections were stained by oil red solution and hematoxylin. After staining, liver sections were mounted by resin. Images were obtained Zeiss microscope (AXIO Observer A1).

### TG Detection

Liver TG was extracted by chloroform: methanol (2: 1) and TG of ZFL cells was extracted by n-hexane: isopropanol (3: 2). Fresh livers were homogenized in 1 mL PBS, then mixed with 5 mL chloroform: methanol (2: 1) vigorously. After standing for 10 min, TG-contained sublayer was extracted and heated with nitrogen at 70°C. ZFL cells were broken by sonication in 1 ml cell lysis, and then mixed with 5 ml n-hexane: isopropanol (3: 2) vigorously. After standing for 10 min, TG-contained uplayer was extracted and heated with nitrogen at 70°C. TG was dispersed by deionized water. The TG content was determined by a quantitative enzymatic method as described in a previous study (35) and was expressed as TG weight per gram protein (mg. g protein^−1^).

### Fatty Acid Extraction and Analysis

Total lipid was extracted from feed and liver freeze-dried powder in chloroform/methanol (2:1, v/v), methylated, and trans-esterified with boron trifluoride in methanol. FAMEs were separated and quantified by GC (Agilent 6890; Agilent, Savage, MD, USA) equipped with a flame ionization detector and a 60.0 m × 250 μm × 0.25 μm fused silica capillary column (DB-23). Helium was used as the carrier gas at a flow rate of 2 ml/min. The temperature was initially 50–220°C at 4°C/min, and then held at 220°C for 35 min. The injector and detector temperatures were set at 260 and 270°C, respectively. Individual methyl esters were identified by comparison to known standards (12, 36).

### DHA and Oleic Acid Solution Preparation

The DHA (D2534, CAS#6217-54-5) and oleic acid (OA, CAS#143-19-1) used in cell culture was purchased from Sigma (USA). A solution of 10 mM DHA and OA were prepared by dilution in 5% fatty acid-free BSA (Sigma, CAS#9048-46-8) and storaged in −20°C. The prepared DHA and OA solutions were subjected to vortexing before use in cell culture.

### Cell Culture and Treatments

The ZFL cell line was purchased from American Type Culture Collection (Manassas, VA, USA), and cultured according to established protocols (37). All media, L-15 (CAS#10045CVR), DMEM (LOT#14318009) and DMEM/F12 (LOT#35220009), were obtained from Corning Inc. (New York, NY, USA). Penicillin-Streptomycin solution (LOT#30002304) and bovine insulin (CAS#11070-73-8) were purchased from Sigma (St. Louis, MO, USA). Murine epidermal growth factor (LOT#0221179) was purchased from Peprotech (Rocky Hill, NJ, USA). Rainbow trout serum (LOT#05191027) was purchased from Caisson Labs (USA). For the detection of cell growth by Real-Time Cell Analyzer (RTCA) xCelligence System (ACEA Biosciences Ins, Santiage, USA), the treatment of DHA was lasting for 48 h. For TG quantification and qPCR analysis, the treatment periods were 24 h.

RTCA xCelligence System (ACEA Biosciences Ins, Santiage, USA) was used to detect real-time growth of ZFL cells treated with different doses of DHA (prepared in 1% BSA) for 48 hrs. ZFL cells were seeded at a density of 3 × 10^4^ cells/well on an E-plate. The treatment started 24 h post-seeding (sub-confluence) by adding DHA to fresh medium at final concentrations of 10 and 50 μM. CI values were monitored in 15 min intervals to 48 h after treatments.

For cell cycle and cell apoptosis analysis, ZFL cells were seeded at a density of 1 × 10^5^ cells/well on a six-well plate. The treatment started 24 h post-seeding by adding DHA to fresh medium at final concentrations of 10 and 50 μM. After 24-h treatment, cells were harvested for cell cycle and cell apoptosis analysis by Guava easyCyte Flow Cytometer (Merck Millipore, Stafford, VA, USA).

For the determination of intracellular TG level and gene expression, ZFL cells were were seeded at a density of 1 × 10^5^ cells/well on a six-well plate and treated with 100 μM OA (OA100) and mixture of 50 μM OA and 50 μM DHA (ODHA) at 24 h post-seeding. After 24-h treatment, cells were harvested for the detection of intracellular TG level and gene expression.

LY2835219 (Selleck) prepared in water, an effective inhibitor of Cyclin D1-CDK4 pathway, was used to block Cyclin D1-CDK4 signal. ZFL cells were seeded at a density of 1 × 10^5^ cells/well on a six-well plate. After 24 h, LY2835219 was added to cell medium at 2 nM. Cells were harvested at 24 h post-treatment. Then expression of genes related to lipid metabolism were detected by real-time fluorescence quantitative PCR (*q*PCR).

### Cell Growth by Real-Time Cell Impedance Analysis

The RTCA xCelligence System (ACEA Biosciences Inc., San Diego, CA, USA) was used to detect real-time cell growth at different doses of DHA. The RTCA software supplied by the manufacturer was used to analyze cell growth and calculate the doubling time of the cells based on the cell index. ZFL cells were seeded at a density of 3 × 10^4^ cells/well on an E-plate. DHA treatment started at 24 h post-seeding. Cell index values were monitored in 15-min interval during 48 h-treatment. The results were plotted as lines by the RTCA software.

### Cell Apoptosis and Cell Cycle Analysis With Flow Cytometry

Cell apoptosis detection was performed with Annexin V-fluorescein isothiocyante (FITC) kits (Sigma, LOT#095M4121V). After exposure to the indicated concentrations of DHA for 24 hrs, cells were collected and incubated with Annexin V-FITC and propidium iodide (PI) in binding buffer in the dark for 10 min at room temperature. The analysis was conducted by the Guava easyCyte Flow Cytometer (Merck Millipore, Stafford, VA, USA). The fluorescence intensity was measured at an excitation wavelength of 488 nm using GRN (525 nm) and RED (690 nm) filters. Data analysis was performed using Flow Guava software.

For cell cycle assay, ZFL cells were fixed with 70% ethanol at 4°C for 24 h and then stained with PI in binding buffer at 37°C in dark for 30 min. Cell cycle was detected by measuring the DNA contents in cells using guava easyCyte flow cytometer. The proportions of cells in the SUBG1, G1, S, and G2/M phases were calculated by Flow guava software. Fluorescence was measured with an excitation wavelength of 488 nm through RED filters (690 nm). Data analysis was performed using Flow Guava software.

### Bodipy Staining

ZFL cells were washed with PBS three times, fixed with 4% paraformaldehyde for 1 hr, and stained with bodipy 493/503 (1 μg / mL in PBS) (Invitrogen, CAS#D3992) in the dark for 15 min at room temperate. The bodipy-stained cells were re-stained with Hochest (1 μg/ml in PBS) (Invitrogen, CAS#H21486) for 5 min following with PBS rinse for three times. Image was acquired with Leica microscope (Leica DMIL-LED).

### Gene Silencing With *si*RNA

Negative control (scrambled *si*RNA, NC), *Cyclin D1 si*RNA (Table 3) were synthesized by GenePharma Co. Ltd. (Shanghai, China). Cells were first seeded on 6-well plates (Corning) and incubated for 24 h to sub-confluence. Then the medium was removed and the cells were transfected with the scrambled *si*RNA and *Cyclin D1 si*RNA using Lipofectamine RNAiMAX Transfection Reagent (Invitrogen, LOT#2098845). The concentration of *si*RNA stock solution was 20 μM. One transfection dispersion for one well consisted of 25 pmol *si*RNA. After 24 h, efficiency of the *si*RNA was determined by *q*PCR.

**Table 3:** Quantitative PCR primers and *si*RNA sequences.

	**Sense (5^′^-3^′^)**	**Antisense (5^′^-3^′^)**
*Rps11*	acagaaatgccccttcactg	gcctcttctcaaaacggttg
*C/ebpα*	gccgcatctgtcctacctt	tgtttcttggatttccctcg
*Srebp 1c*	cagagggtgggcatgctggc	atgtgacggtggtgccgctg
*Acc1*	gcgtggccgaacaatggcag	gcaggtccagcttccctgcg
*Fas*	ggagcaggctgcctctgtgc	ttgcggcctgtcccactcct
*Scd1*	gcttttgcgtgtttcgtgta	ggtttgagttgtgagggtcg
*Dgat2*	ccttacacgacctgcccac	caaaaagccccaaaacacaa
*Pparα*	gatattagatgtcttaactccggc	cagtttaagtcgaatggttctc
*Acox1*	agcacagcaagagtaacgaaga	ggcataaagcagagccaaag
*Lcad*	gtggtcctggcttctctctcc	tcagttccatccttcttcgca
*Mcad*	cagaaagagttccaggaggtg	tgtccgttcattagacccag
*Vlcad*	cacaggtctttccctatccc	agagcatcgttcttcatcgg
*Cpt2*	gaccccaaacccgaatac	tgagttgaagaggcgaaagt
*Cyclin D1*	gttttgctgcgaagtgga	Cctggtttttttggtggg
*Cyclin D2a*	gttcagaaagacattcagccct	ggtccaagtagttcatagccag
*Cyclin D2b*	ggatgctggaggtgtgtga	atgaaggtttgcgtgtgct
*Cyclin D3*	cgaacatcagcggaaaaca	tccaggaagtcagagggca
*Cyclin D1* siRNA	ggaaucgagucucagacaatt	uugucugagacucgauucctt

### Gut Microbiota Analysis

The 16s V3–V4 region was amplified using the primer pairs 338F (5′-ACTCCTACGGGAGGCAGCAG-3′) and 806R (5′-CCATTGTAGCACGTGTGTAGCC-3′). The 16S ribosomal RNA gene sequencing was performed by Majorbio Bio-Pharm Technology Co. Ltd. (Shanghai, China) using Illumina MiSeq PE300 platform (Illumina, San Diego, USA). Then the raw pair-end readings were subjected to a quality-control procedure using the UPARSE-operational taxonomic unit (OTU) algorithm (38). The qualified reads were clustered to generate OTUs at the 97% similarity level using the USEARCH sequence analysis tool (38). A representative sequence of each OTU was assigned to a taxonomic level in the Ribosomal Database Project (RDP) database using the RDP classifier (39).

### Germ-Free Zebrafish Culture and Treatment

GF zebrafish were generated from normal parent zebrafish through disinfecting with antibiotics, PVP and sodium hypochlorite in sequence, and reared following established protocols (40). Zebrafish larvae hatched from their chorions at 3 days postfertilization (dpf). Each group had six bottles with 20 fish per bottle. At 5 dpf, the yolk was largely absorbed and the GF-zebrafish was subjected with feeding and transferring trials. In the feeding trial, GF zebrafish were fed with LFD, HFD and HFDHA0.5 (Table 4). All dry ingredients were ground through a 100-mesh screen. The diets were prepared by mixing the dry ingredients with the oil and water manually. Then each diet was extruded in a manual extruder with a 2.5-mm aperture. The extruded pellets were freeze-dried and stored at −20°C in plastic bags in small quantities. Before sterilization, the feed pellets were ground through a 60-mesh screen. The diets for GF zebrafish were sterilized by irradiation with 20 kGy gamma ray (Institute of Food Science and Technology, Chinese Academy of Agricultural Sciences, Beijing, China).

**Table 4:** Ingredients of experimental diets for germ-free zebrafish (g/kg).

**Ingredients (g/kg dry diet)**	**Zebrafish larva**		
	**LFD**	**HFD**	**HFDHA0.5**
Casein	460	460	460
Geltin	110	110	110
Wheat flour	280	120	120
Soybean oil	35	160	155
Cod liver oil	35	40	40
DHA^a^	0	0	5
Soybean lecithin	20	20	20
Lysine	3.7	3.7	3.7
VC phosphate	1	1	1
Vitamin premix^b^	2	2	2
Mineral premix^c^	2	2	2
Monocalcium phosphate	20	20	20
Choline chloride	2	2	2
Sodium alginate	20	20	20
Microcrystalline cellulose	9.3	39.3	39.3
Total	1,000	1,000	1,000

a *Larodan*.

b *Vitamin premix (g/kg): thiamine, 0.438; riboflavin, 0.632; pyridoxine·HCl, 0.908; d-pantothenic acid, 1.724; nicotinic acid, 4.583; biotin, 0.211; folic acid, 0.549; vitamin B-12, 0.001; inositol, 21.053; menadione sodium bisulfite, 0.889; retinyl acetate, 0.677; cholecalciferol, 0.116; dl-α-tocopherol-acetate, 12.632*.

c *Mineral premix (g/kg): CoCl_2_·6H_2_O, 0.074; CuSO_4_·5H_2_O, 2.5; FeSO_4_·7H_2_O, 73.2*.

In the transferring trial, GF zebrafish were transferred with gut microbiota deprived from conventional zebrafish fed LFD, HFD and HFDHA0.5. At 12 dpf, GF zebrafish in the feeding trial were collected for for oil red staining and *q*PCR. At 8 dpf, zebrafish larvae transferred with LFD, HFD and HFDHA0.5-gut microbiota were collected for oil red staining and *q*PCR.

### Total RNA Extraction, Reverse Transcription, and *Q*PCR

Total RNA was isolated using Trizol reagent (LOT#03877/35120) (Cwbio, Beijing, China) and then reversed transcribed to cDNA by FastKing gDNA Dispelling RT SuperMix (LOT#W9813) (Tiangen, Beijing, China). The *q*PCR was performed using SYBR^®^Green Supermix (LOT#W9413) according to the manufacturer's instructions (Tiangen, Beijing, China). The results were stored, managed, and analyzed using LightCycler 480 software (Roche, Basel, Switzerland). The *q*PCR primers used are listed in Table 3.

### Data Analysis

The statistical analyses were conducted using GraphPad Prism 5 softw4are (GraphPad Software Inc., San Diego, CA, USA). Results are expressed as the means ± standard errors of the means (SEMs). Comparisons between two groups were analyzed using the Student's *t*-test, and comparisons between multiple groups were analyzed using one-way ANOVA followed by a Duncan's test. The statistical significance was set at *P* < 0.05 and *P* < 0.01.

## Results

### The Effects of DHA on Survival, Body Weight Gain, Feed Efficiency

**Figure 1:**
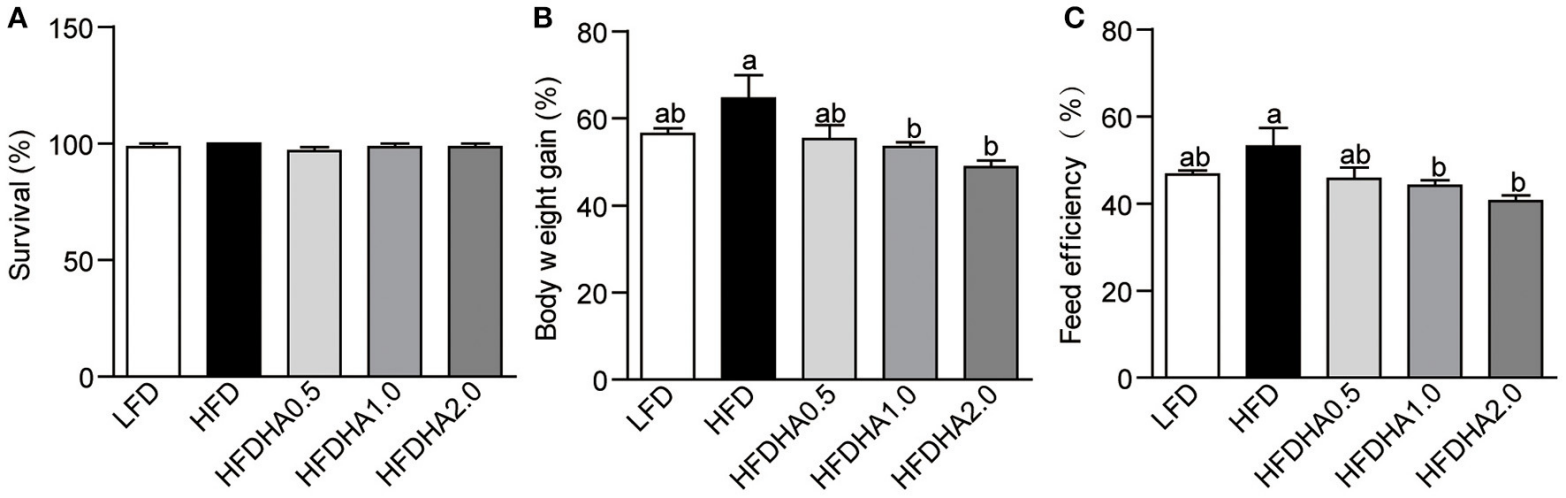
Effects of DHA supplementation on **(A)** Survival, **(B)** body weight, **(C)** feed efficiency. Values are means ± SEMs (*n* = 3 biological replicates). Means without a common letter are significantly different, *P* < 0.05.

At the end of 2-wk feeding trial, survival was similar among groups (Figure 1A). The body weight gain in HFD-fed zebrafish was moderately higher than that of fish fed LFD (64.56 vs. 54.44%; Figure 1B). Zebrafish fed HFDHA0.5 had moderate lower body weight gain than that in zebrafish fed HFD (55.21 vs. 64.56%) (Figure 1B). However, zebrafish fed HFDHA1.0 and HFDHA2.0 had significantly lower body weight gain as compared to those fed HFD (53.42, 48.77 vs. 64.56%, respectively; *P* < 0.05; Figure 1B). The feed efficiency of zebrafish fed HFDHA0.5, HFDHA1.0, and HFDHA2.0 were 45.65, 44.16, and 40.52%, respectively (Figure 1C). Consistently, the feed efficiency in HFDHA0.5 group was non-significantly lower than that in HFD group (Figure 1C). The feed efficiency in HFDHA1.0 and HFDHA2.0 groups were significantly lower than that in HFD group (*P* < 0.05; Figure 1C). These results suggested that the supplementation of DHA to HFD can inhibit fish growth, especially at the additive amount higher than 0.5%.

### The Effects of DHA on Hepatic Lipid Metabolism

**Figure 2:**
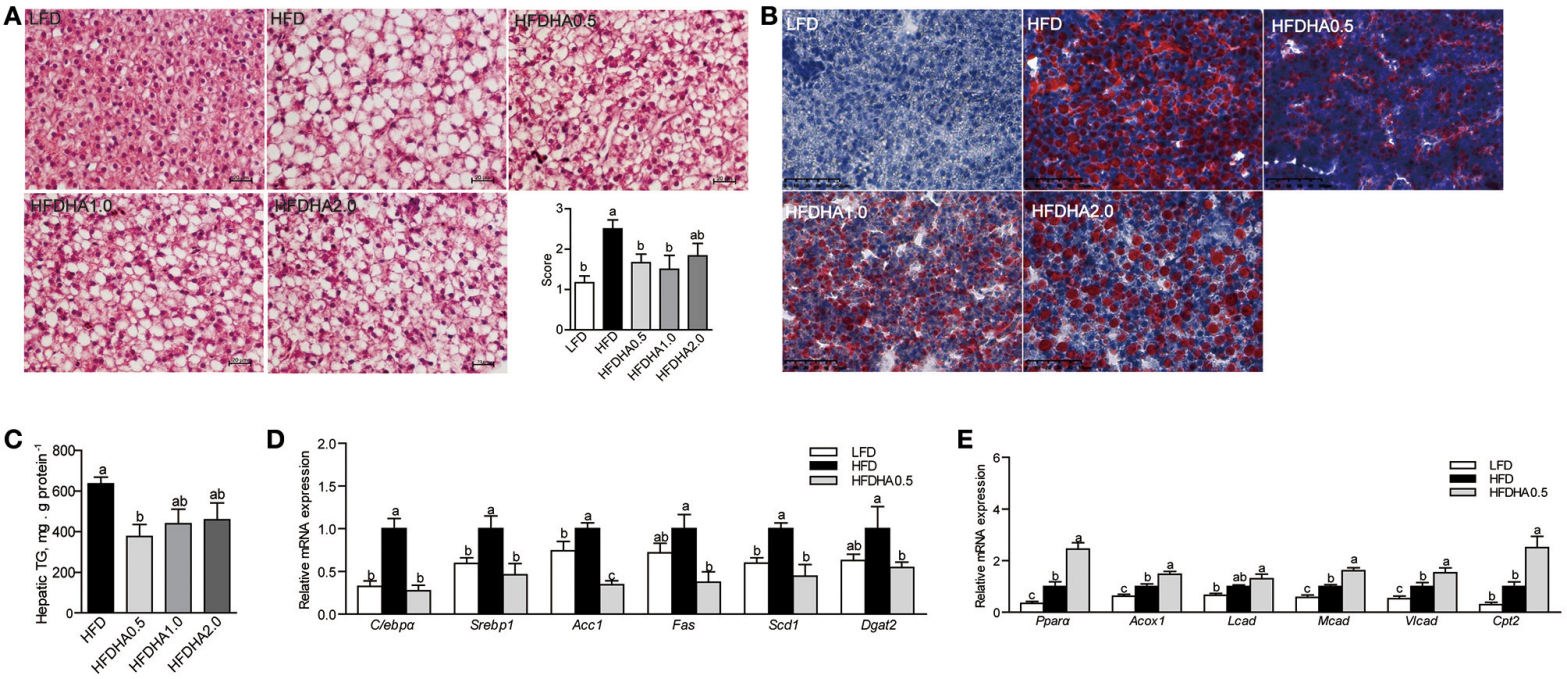
Effects of DHA supplementation on hepatic lipid metabolism. **(A)** H&E staining of liver sections of LFD, HFD, and HFDHAs-fed zebrafish for 2 wks. The scale bar is 20 μm. **(B)** Oil red staining of liver sections of LFD, HFD, and HFDHAs-fed zebrafish for 2 wks. The scale bar is 50 μm. **(C)** Hepatic TG content of zebrafish fed HFD and HFDHAs for 2 wks. Relative mRNA expression of genes related to **(D)** lipid synthesis and **(E)** β-oxidation. Values are means ± SEMs (*n* = 3-5 biological replicates). Means without a common letter are significantly different, *P* < 0.05.

Zebrafish fed HFD developed a large vesicular intracellular lipid droplets as revealed by H&E staining and oil red staining (Figures 2A,B). Zebrafish fed HFDHA0.5, HFDHA1.0, and HFDHA2.0 showed a tendency of alleviated hepatic steatosis as compared to those fed HFD (Figures 2A,B). However, only HFD supplemented with 0.5% DHA significantly led to a 40.8% reduction in hepatic TG level (*P* < 0.05; Figure 2C). Thus, only the 0.5% DHA supplementation was used for the further analysis in this study. This also appeared in a 4-wk feeding trial (Supplementary Figure 1).

Relative to LFD, feeding HFD significantly upregulated liver mRNA expression of CCAAT/enhancer binding protein α (*C/ebp*α), sterol regulatory element binding transcription factor 1 (*Srebp1*), acetyl-CoA carboxylase (*Acc1*), stearoyl-Coenzyme A desaturase 1 (*Scd1*) and diacylglycerol O-acyltransferase 2 (*Dgat2*) (*P* < 0.05; Figure 2D). When the fish were fed the HFDHA0.5 and compared to the HFD, the relative mRNA expressions of *C/ebp*α, *Srebp1, Acc1, Fas, Scd1*, and *Dgat2* in liver were reduced and for most parts similar to that of LFD-fed fish (*P* < 0.05; Figure 2D). Feeding HFD led to a small increase in peroxisome proliferator activated receptor α (*Ppar*α), acyl-CoA oxidase 1 (*Acox1*) and medium-chain acyl-CoA dehydrogenase (*Mcad*), but not long-chain acyl-CoA dehydrogenase (*Lcad*), carnitine palmitoyltransferase 2 (*Cpt2*) when compared to those fed LFD (Figure 2E), however, feeding HFDAH0.5 led to massive increases in the relative mRNA expression of all these genes (*P* < 0.05; Figure 2E). Although the total hepatic PUFA content in HFD and HFDHA0.5 groups showed no significant difference, the concentration of *n* – 3 PUFA in HFDHA0.5 group was 46.7% higher than that in HFD group (*P* < 0.01; Table 5). Moreover, zebrafish fed HFDHA0.5 had higher hepatic DHA level than fish fed HFD (*P* < 0.05; Table 5). These results suggested that the supplementation of 0.5% DHA can regulate hepatic lipid metabolism partly by inhibiting hepatic lipid synthesis and promoting lipid β-oxidation, and increase the accumulation of DHA in liver.

**Table 5:** Fatty acid composition in the liver of 1-month-old zebrafish fed diets containing DHA for 2-wk.

**g/100 g total fatty acid**	**HFD**	**HFDHA0.5**
C14:0	0.63 ± 0.05	0.67 ± 0.00
C16:0	19.69 ± 0.23	14.70 ± 0.13^**^
C18:0	8.33 ± 0.80	8.77 ± 0.05
C21:0	0.64 ± 0.01	0.69 ± 0.00
Total saturates^1^	30.21 ± 1.15	25.74 ± 0.08^*^
C18:1	32.54 ± 0.95	33.23 ± 0.15
Total monoenes^2^	33.03 ± 0.99	33.71 ± 0.16
C18:2	26.77 ± 2.73	27.82 ± 0.02
C20:3	1.96 ± 0.19	2.79 ± 0.02^*^
C20:4	2.95 ± 0.43	2.49 ± 0.00
Total (*n* – 6)^3^	31.68 ± 2.49	33.10 ± 0.04
C18:3	1.97 ± 0.02	2.46 ± 0.03^**^
C20:5	0.66 ± 0.06	0.69 ± 0.01
C22:6	2.32 ± 0.38	4.16 ± 0.06^*^
Total (*n* – 3)^4^	5.08 ± 0.35	7.45 ± 0.03^*^
Total PUFA	36.76 ± 2.14	40.55 ± 0.08
(*n* – 3):(*n* – 6)	0.16	0.22

*Values are means ± SEMs; n = 3*.

* *P < 0.01*,

** *P < 0.05*.

1 *includes 6:0, 10:0, 12:0, 14:0, 15:0, 16:0, 17:0, 18:0, 20:0, 21:0, 22:0, 24:0*.

2 *includes 14:1, 16:1, 18:1, 20:1, 22:1, 24:1*.

3 *includes 18:2, 20:3, 20:4*.

4 *includes 18:3, 20:3, 20:5, 22:6*.

*LFD, low-fat diet; HFD, high-fat diet; HFDHA, DHA-supplemented HFD*.

We also confirmed the effect of dietary DHA on muscle, the largest part of fish body. The muscular TG level and the mRNA expression of *Scd1* and *Dgat2* were non-significantly reduced while muscular *Acox1* and *Cpt2* were non-significantly increased in HFDHA0.5 group as compared to HFD group (Supplementary Figures 2A–C). Thus, 0.5% DHA supplementation had no significant effect on muscle lipid metabolism.

### DHA Modulates Lipid Metabolism in ZFL Cell Model

**Figure 3:**
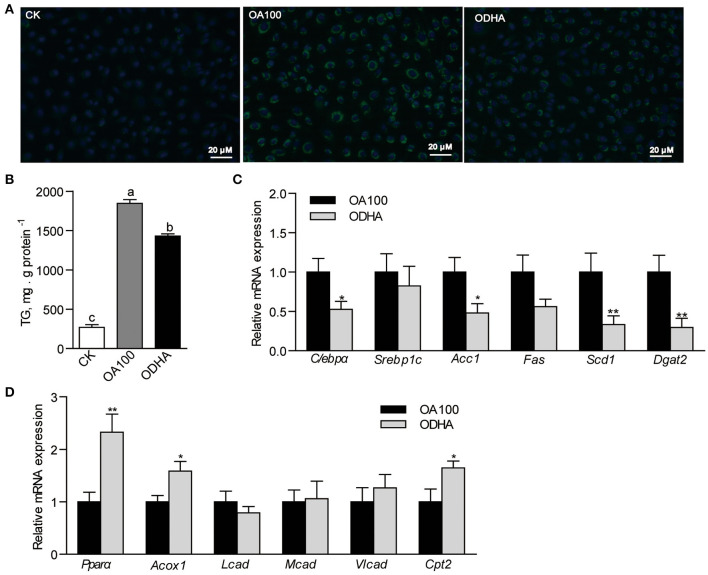
Modulation of DHA on lipid metabolism in ZFL cells. **(A)** Lipid droplets accumulation in ZFL cells with lipid treatment. The scale bar is 20 μm. **(B)** TG content in ZFL cells treated with CK, OA, and ODHA. **(C)** Relative mRNA expression of genes related to lipid synthesis and **(D)** β-oxidation in control and lipid treated ZFL cells. Values are means ± SEMs (*n* = 5-6 biological replicates). Means without a common letter are significantly different, *P* < 0.05. *P < 0.05; **P < 0.01. CK, controls; OA, 100 μM oleic acid; ODHA, mix of 50 μM OA and 50 μM DHA.

The results showed that the intracellular lipid droplets, marked by the green fluorescencent probe BODIPY 493/503, were reduced in cells treated with ODHA (Figure 3A). Moreover, the TG content in ZFL cells treated with ODHA was 22.6% lower than that in OA100-treated cells (*P* < 0.05; Figure 3B). The relative mRNA expression of lipid synthesis-related genes (*C/ebp*α, *Scd1* and *Dgat2*) in cells treated with ODHA were significantly lower than that in OA100-treated cells (0.53-, 0.33-, and 0.30-fold; *P* < 0.05; Figure 3C), whereas the relative mRNA expressions of the fatty acid β-oxidation-related genes *Ppar*α, *Acox1*, and *Cpt2* were significantly higher than that in OA100-treated cells (2.33-, 1.59-, and 1.65-fold; *P* < 0.05; Figure 3D). These results indicated that DHA can also regulate lipid metabolism by inhibiting lipid synthesis and promoting β-oxidation *in vitro*.

### DHA Modulates *Cyclin D1* Expression in Liver

**Figure 4:**
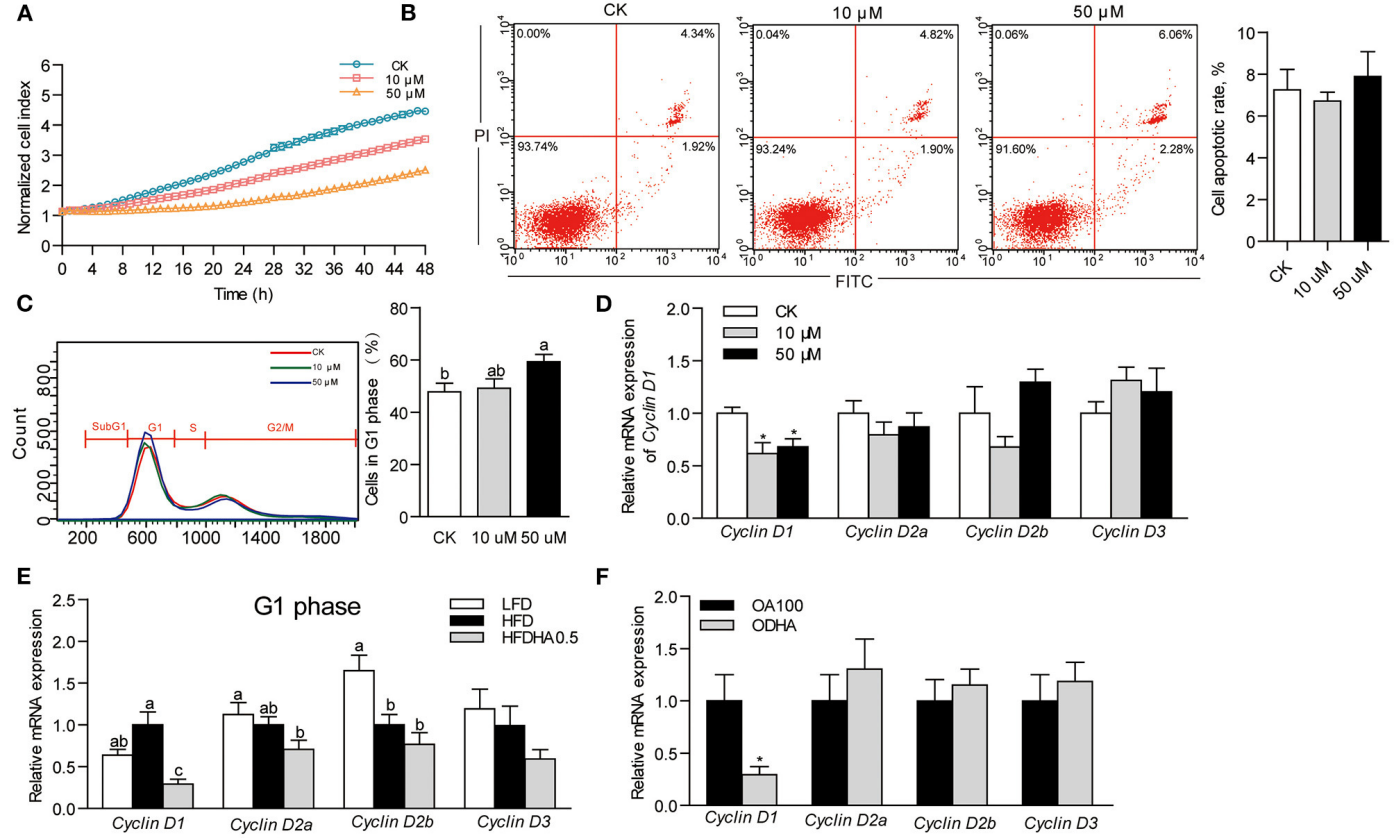
Effects of DHA on cell growth, cell apoptosis and cell cycle. **(A)** Growth of ZFL cells treated with DHA for 48 h. **(B)** Cell apoptosis and **(C)** cell cycle of ZFL treated with DHA at 24 h. **(D)** Relative mRNA expression of genes encoding D-type Cyclins in ZFL cells. **(E)** Relative mRNA expression of genes encoding D-type Cyclins in the liver of LFD, HFD, and HFDHA0.5-fed zebrafish. **(F)** Relative mRNA expression of genes encoding D-type Cyclins in ZFL cells treated with OA and ODHA. Values are means ± SEMs (*n* = 3-5 biological replicates). Means without a common letter are significantly different, *P* < 0.05. *P < 0.05.

Results showed that DHA inhibited cell growth in a concentration-dependent manner (Figure 4A). At 24 h, the growth of ZFL cells treated with 10 and 50 μM DHA was inhibited by 24.5 and 51.4%, respectively (Figure 4A). However, DHA did not induce cell apoptosis (Figure 4B). Flow cytometry analysis of the cell cycle indicated that 50 μM DHA arrested ZFL cells in the G1 phase at 24 h (Figure 4C). The relative mRNA expression of *Cyclin D1* in 10 and 50 μM DHA-treated ZFL cells were significantly reduced as compared to control cells, respectively (*P* < 0.05; Figure 4D).

Accordingly, we analyzed cell cycle by analyzing the expression of genes encoding cell cycle modulators of zebrafish liver. Changing from LFD to HFD had little effect on the mRNA expression of the G1 phase-related modulators *Cyclin D1, Cyclin D2a and Cyclin D3* but reduced the expression of *Cyclin D2b* (Figure 4E). Replacing HFD to HFDHA0.5 led to a clear tendency of further reduction of all genes but was only significant for *Cyclin D1* (0.29-fold; *P* < 0.05; Figure 4E). The relative mRNA expression of the G2 phase-related modulator, Cyclin B, was similar between zebrafish fed HFD and HFDHA0.5 (Supplementary Figure 3A). Besides, the relative mRNA expression of *Cyclin D1* in ODHA-treated ZFL cells was significantly reduced as compared to that in OA100-treated cells (0.29- fold; *P* < 0.05; Figure 4F). These results indicated that DHA can induce cell cycle arrest at G1 phase both in liver tissue and *in vitro* cell model.

### The Suppression of Cyclin D1 Modulates Lipid Metabolism in ZFL Cell Model

**Figure 5:**
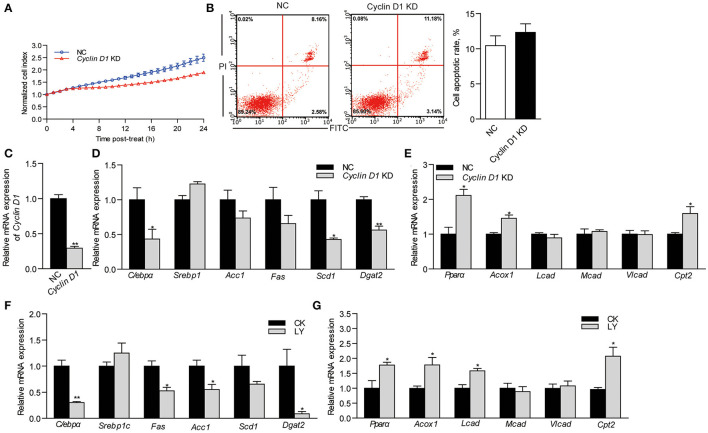
Effects of *Cyclin D1* knockdown and Cyclin D1-Cdk4 blocking on lipid metabolism. **(A)** Growth of NC and Cyclin D1-knockdown ZFL cells. **(B)** Apoptosis of NC and Cyclin D1-knockdown ZFL cells. **(C)** Efficiency of *si*RNA targeting *cyclin D1* in ZFL cells. **(D)** Relative mRNA expression of genes related lipid synthesis in ZFL cells with *Cyclin D1* knockdown. **(E)** Relative mRNA expression of genes related β-oxidation in ZFL cells with *Cyclin D1* knockdown. Relative mRNA expression of genes related **(F)** lipid synthesis and **(G)** lipid β-oxidation in ZFL cells treated with LY2835219. Values are means ± SEMs (*n* = 3 biological replicates). *P < 0.05; ^**^P < 0.01.

To determine the relationship between *Cyclin D1* and lipid metabolism, we repressed the expression of *Cyclin D1* by transfecting *si*RNA into ZFL cells. The growth of *Cyclin D1*-knockdown ZFL cells was slower than that in NC cells (Figure 5A). However, *Cyclin D1* knockdown showed no significant influence on cell apoptosis (Figure 5B). The results showed that *si*RNA of *Cyclin D1* reduced its mRNA expression by 70.7% as compared to NC (*P* < 0.05; Figure 5C). The expression of the lipid synthesis-related genes (*C/ebp*α, *Scd1*, and *Dgat2*) in *Cyclin D1*-knockdown ZFL cells were significantly lower than that in NC-treated ZFL cells (0.43-, 0.43- and 0.56-fold; *P* < 0.05; Figure 5D), while the relative mRNA expression of the fatty acid β-oxidation-related genes *Ppar*α, *Acox1*, and *Cpt2* were significantly higher (2.11-, 1.45-, and 1.59-fold; *P* < 0.05; Figure 5E). Furthermore, when we used the Cyclin D1-Cdk4 inhibitor, LY2835219, to induce cell arrest in G1 phase, we observed decreased expression of genes related to lipid synthesis (*C/ebp*α, *Scd1, Acc1*, and *Dgat2*) (Figure 5F) while the expression of genes related to lipid β-oxidation (*Ppar*α, *Acox1*, and *Cpt2*) increased (Figure 5G). These results suggested that *Cyclin D1* and Cyclin D1-Cdk4 signal were involved with, at least partly, the modulation of genes expression involved in lipid metabolism.

### Dietary DHA Directly Modulates Lipid Metabolism in Germ-Free Zebrafish Model

**Figure 6:**
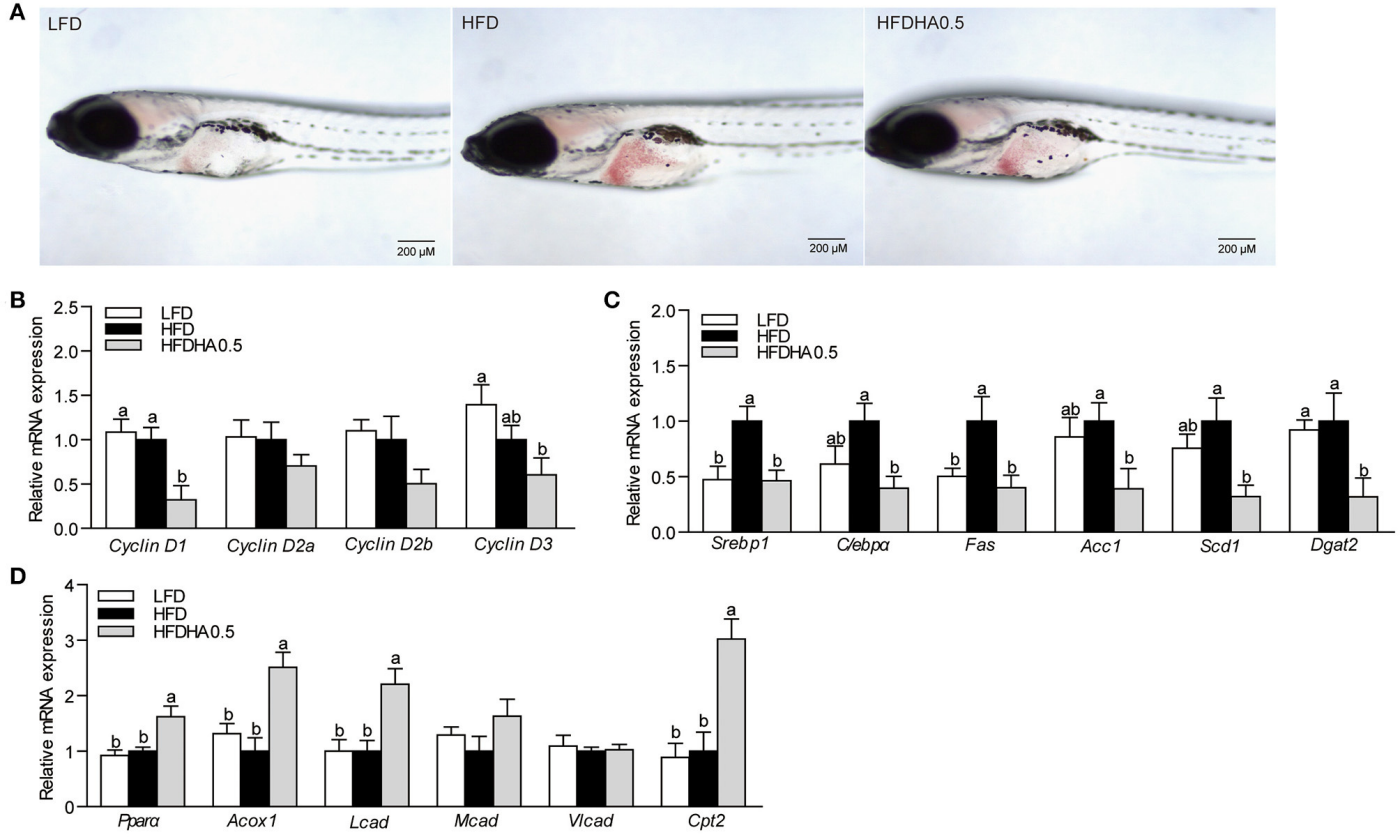
Effects of DHA supplementation on lipid metabolism in GF zebrafish. **(A)** Whole-mount oil red staining in GF zebrafish fed LFD, HFD and HFDHA0.5. The scale bar is 200 μm. **(B)** Relative mRNA expression of CyclinDs. Relative mRNA expression of **(C)** lipid synthesis-related, and **(D)** lipid β-oxidation-related genes. Values are means ± SEMs (*n* = 4 biological replicates). Means without a common letter are significantly different, *P* < 0.05.

To investigate the effects of dietary DHA with no involvement of gut microbiota, HFDHA0.5 was fed to GF-zebrafish larvae. After feeding for 1-wk, HFDHA0.5-fed GF zebrafish had reduced hepatic steatosis compared to HFD-fed zebrafish (Figure 6A) and reduced relative mRNA expression of *Cyclin D1* (*P* < 0.05; Figure 6B). Furthermore, the relative expression of the lipid synthesis-related genes tested were significantly lower in the HFDHA0.5 group compared to those fed HFD (*P* < 0.05; Figure 6C), while the expression of some lipid β-oxidation-related including *Ppar*α, *Acox1* and *Cpt2* were upregulated (*P* < 0.05; Figure 6D). These results suggested that DHA can directly modulate lipid metabolism in zebrafish.

### Dietary DHA Supplementation Leads to Gut Microbial Community Changes in Zebrafish

**Figure 7:**
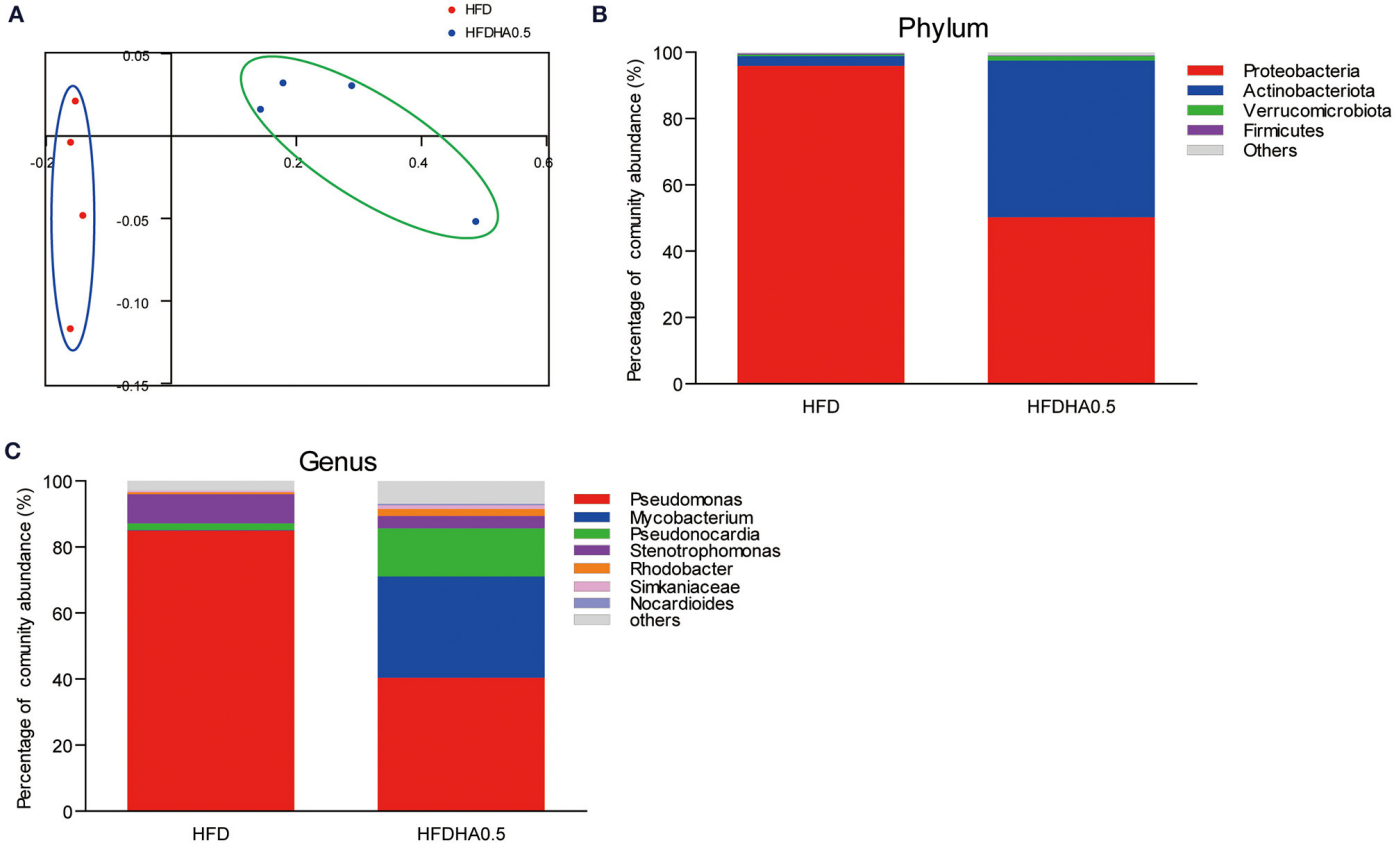
Effects of DHA supplementation on gut microbial community in zebrafish. **(A)** PCoA analysis of gut microbiota in HFD- and HFDHA0.5-fed zebrafish. **(B)** Relative abundance at the phylum level of gut microbial community from HFD- and HFDHA0.5-fed zebrafish **(C)** Relative abundance at the genus level of gut microbial community from HFD- and HFDHA0.5-fed zebrafish. Values are means ± SEMs (*n* = 4 biological replicates).

At the end of the 2-wk feeding trial, we analyzed the effects of DHA supplementation on the gut microbial community. The results clearly showed that adding DHA to the HFD significantly altered the composition as shown through clustering analysis in Figure 7A. *Proteobacteria* (95.9%) was the dominant phylum in HFD-fed zebrafish (Figure 7B), while *Proteobacteria* (50.2%) and *Actinobacteriota* (47.3%) were the predominant phylums in HFDHA0.5-fed zebrafish (Figure 7B). Relative abundance analysis at the genus level showed significant enrichment of *Mycobacterium* in the HFDHA0.5 group, whereas the abundance of *Pseudomonas* was significantly reduced in the HFDHA0.5 group as compared to the HFD group (*P* < 0.05; Figure 7C; Table 6). The diversity indexes of the gut microbial community including Shannon-, Ace- and Chao-indexes were higher in the HFDHA0.5 (*P* < 0.05), while the Simpson-index was lower (*P* < 0.05) that in HFD group (Table 7).

**Table 6:** The predominant gut bacterial genus in zebrafish fed the LFD, HFD, or HFDHA 0.5 for 2 weeks based on V3–V4 sequences.

**Genus (%)**	**LFD**	**HFD**	**HFDHA0.5**
*Pseudomonas*	84.00 ± 1.46^a^	84.98 ± 1.09^a^	40.35 ± 6.80^b^
*Mycobacterium*	0.04 ± 0.02^b^	0.11 ± 0.02^b^	30.73 ± 6.86^a^
*Pseudonocardia*	1.95 ± 2.03	2.03 ± 1.25	14.51 ± 9.76
*Stenotrophomonas*	8.18 ± 0.31^a^	8.80 ± 0.21^a^	3.81 ± 0.76^b^
*Rhodobacter*	0.21 ± 0.16^b^	0.59 ± 0.26^b^	2.09 ± 0.54^a^
*Simkaniaceae*	0.95 ± 0.17	0.50 ± 0.32	1.12 ± 0.53
*Nocardioides*	1.74 ± 0.62^a^	0.04 ± 0.01^b^	0.47 ± 0.13^b^

*Values are expressed as the mean ± SEM, n = 4. Means marked with different letters represent statistically significant results (P < 0.05), whereas the same letter correspond to results that show no statistically significant differences*.

**Table 7:** Diversity index of gut bacteria of zebrafish fed with the LFD, HFD or HFDHA0.5 for 2 weeks.

	**LFD**	**HFD**	**HFDHA0.5**
Shannon	0.74 ± 0.08^b^	0.71 ± 0.05^b^	1.68 ± 0.11^a^
Simpson	0.71 ± 0.02^a^	0.73 ± 0.02^a^	0.32 ± 0.03^b^
Ace	207.02 ± 23.81^b^	220.51 ± 20.35^ab^	278.09 ± 22.44^a^
Chao	209.40 ± 23.04^b^	216.01 ± 13.99^ab^	268.81 ± 21.59^a^

*Values are expressed as the mean ± SEM, n = 4. Means marked with different letters represent statistically significant results (P < 0.05), whereas the same letter correspond to results that show no statistically significant differences*.

### DHA-Altered Gut Microbiota Contribute to Lipid Oxidation in Germ-Free Zebrafish

**Figure 8:**
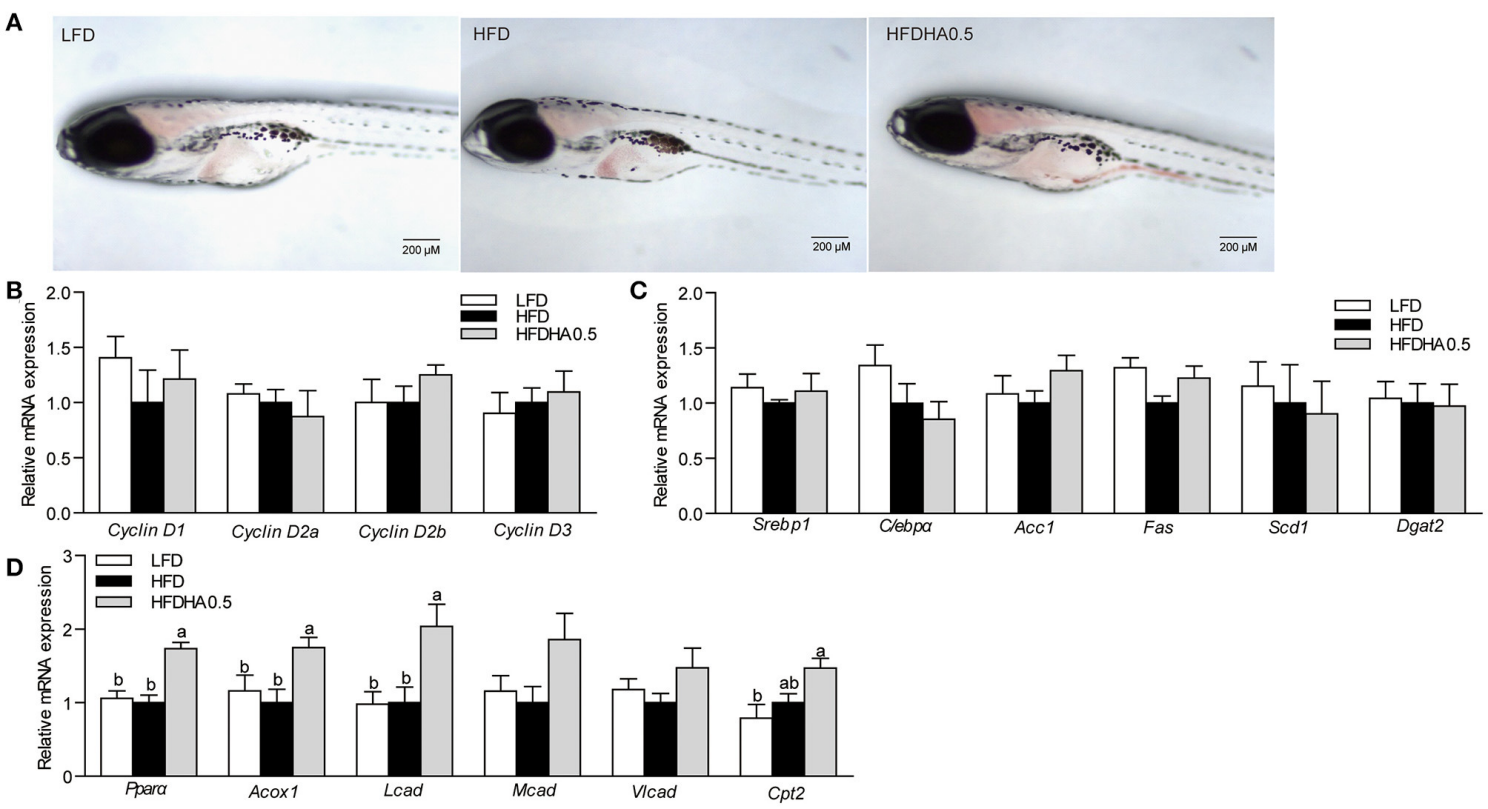
Effects of gut microbiota on lipid metabolism in GF zebrafish. **(A)** Whole-mount oil red staining in GF zebrafish in LFD, HFD, and HFDHA0.5. The scale bar is 200 μm. **(B)** Relative mRNA expression of CyclinDs. Relative mRNA expression of **(C)** lipid synthesis-related, and **(D)** lipid β-oxidation-related genes. Values are means ± SEMs (*n* = 4 biological replicates). Means without a common letter are significantly different, *P* < 0.05.

To investigate the microbiota-mediated effects, gut microbiota from the HFD and HFDHA0.5 groups were transferred to GF-zebrafish. HFDHA0.5 microbiota-colonized GF zebrafish showed lower hepatic steatosis than HFD microbiota-colonized zebrafish (Figure 8A). The colonization of HFDHA0.5-microbiota for 72 h showed no effect on the expression of *Cyclin D*s (*Cyclin D1, D2a, D2b*, and *D3*) (Figure 8B) and genes related to lipid synthesis (Figure 8C), but led to higher expression of the lipid β-oxidation-related genes *Ppar*α and *Acox1*(*P* < 0.05; Figure 8D) as compared to HFD-microbiota. These results showed that the gut microbial community induced by DHA-supplemented diet can modulate lipid metabolism by promoting lipid β-oxidation independent of Cyclin D1.

## Discussion

In this study, the short-term feeding (2 wks) of HFD induced hepatic fat accumulation in zebrafish. In line with these studies in mammals, the current study clearly showed that DHA can reduce the hepatic lipid content in HFD-fed zebrafish. In mammals, DHA is known to have several positive effects of diseases related to fatty liver (14–16). DHA reduces lipid content by downregulating fatty acid synthesis and increase fatty acid β-oxidation in non-alcoholic fatty liver disease (NAFLD) models (19, 20). The mechanism appears in part to be regulated through reduction of the transcription factor SREBP1c, which leads to a downstream reduction of the activity of lipid synthesis genes like *Fas, Scd1, Dgat*s, and an upregulation of *Ppar*α which stimulates peroxisomal and mitochondrial lipid transport and β-oxidation (e.g., *Acox and Cpt2)* (15, 41, 42).

To determine the effects of DHA supplementation on hepatic lipid metabolism, we analyzed the expression of genes related to lipid synthesis and β-oxidation. In this study, the transcription factor for lipid synthesis*, C/ebp*α, was reduced along with genes in the TG synthesis pathway, while *Ppar*α was upregulated along with β-oxidation genes. Besides, the effects of DHA were also confirmed in ZFL cells. The results showed that the modulation of DHA on hepatic lipid accumulation in zebrafish appears to be similar to that in mammals. Moreover, we treated ZFL cells with 100 μM OA and ODHA and found that the partially replacing OA with DHA led to reduced lipid accumulation with decreased lipid synthesis and decreased β-oxidation. However, it is well-known that DHA is relatively resistant to β-oxidation (43, 44) and frequently incorporates to membranes in the form of phospholipids (27, 45). This means DHA might not be the main substrate for β-oxidation. Thus, the increased capacity of β-oxidation induced by DHA might increase the turnover of other obesogenous fatty acids, such as OA. Taken together, these results confirmed a conservative lipid-lowering effect of DHA in zebrafish.

DHA is known to have effects on cell growth and proliferation (28). The increased DHA incorporation into the phospholipids of the nucleus membrane may produce a direct influence on modulation of genes, RNA synthesis and release, and the activation of DNA polymerase α, all that can affect cell proliferation (46). Thus, the effects of DHA on cellular processes including growth, apoptosis and proliferation in zebrafish liver cells (ZFL) were examined after treating ZFL cells with 0, 10, and 50 μM DHA up to 48 h. In the present study, we convincingly showed that DHA inhibited cell growth and led to cell cycle arrest by increasing the number of cells in the G1 phase and reducing mRNA expression of *Cyclin D1*. This agrees with previous reports in both fish and mammals where DHA induced cell cycle arrest *via* Cyclin-Cdk manners (34, 47, 48).

Moreover, Cyclin D1 has its own functions in modulating lipid metabolism *via* its regulation of nuclear receptors and transcriptional modulation (49–53). The direct role of dietary DHA was also tested in the GF zebrafish model where dietary DHA not only inhibited the expression of *Cyclin D1*, but also modulated lipid metabolism similar to that found in the conventional feeding trial. To determine the relationship between *Cyclin D1* and lipid metabolism, we repressed the expression of *Cyclin D1* by transfecting *Cyclin D1 si*RNA into ZFL cells. In *Cyclin D1-*knockdown ZFL cells, we observed that lipid synthesis was inhibited and lipid β-oxidation was promoted. Moreover, the inhibitor for Cyclin D1-Cdk4, LY2835219, exerted similar effects. These results showed that DHA might reduce hepatic lipid accumulation *via* blocking of the Cyclin D1-Cdk4 signal.

Curiously, this appeared to partly contradict to previous studies where Cyclin D1 knockdown was shown to promote hepatic lipogenesis in a well-differentiated AML12 cell line cultured at high density (52). But some indirect evidences suggested the positive role of Cyclin D1 in promoting fatty liver development. The inhibition of Cdk4, the downstream kinase of Cyclin D1, can prevent the development of hepatic steatosis by reducing C/ebpα phosphorylation and its target gene, *Fasn, Acc, Scd, Gpat, Dgat1*, and *Dgat2* (54), which is in line with the effect of LY2835219 (inhibitor for Cyclin D1-Cdk4) in this study. Cell cycle arrest in G1 phase significantly attenuates Srebp-mediated lipid synthesis (55). Due to the scarcity of Cyclin D1 regulatory mode, further studies are needed to fully illustrate the sophisticated mechanism upon Cyclin D1-mediated lipid metabolism. It nevertheless appears quite clear that there is a link between the cell cycle machinery and the regulation of hepatic lipid metabolism.

In our previous studies, HFD could lead to alteration in gut microbial community and reduced diversity in zebrafish (35, 56), which also was observed in this study. In addition, DHA supplementation partly diminished the effects of HFD on the gut microbial community and increased diversity of species. This result was partly in agreement with studies in mice and zebrafish, where *n*–3 PUFA or fish oil supplementation reduced the growth of *Pseudomonas* (57, 58). The formation of biofilm might play an important role in *Pseudomonas's* adherence and growth on any surface, including gut mucosa (59). Although the effect of DHA on *Pseudomonas* biofilm has not been studied, it is clear that DHA can disturb biofilm formation in *Streptococcus* mutans and inhibit its growth (60). Thus, the reduction of *Pseudomonas* caused by DHA supplementation might be due to the anti-bacterial effect mediated by biofilm damage. However, what out of expectation was that DHA supplementation also increased the growth of *Mycobacterium*. *Mycobacterium* is the pathogen causing mycobacteriosis in zebrafish and the gastrointestinal tract is the primary route of infection (61). Although some *Mycobacterium* spp isolated from zebrafish do not cause significant mortalities, granulomatous lesions in visceral organs have been reported in zebrafish (62). No significant mortality was observed in the short-term feeding trial, but further studies are needed to reveal the presence or not of granulomatous lesions in visceral organs so that the effect of increased *Mycobacterium* could be defined.

Thus, to investigate the microbiota-mediated effects on lipid metabolism, gut microbiota from the HFD and HFDHA0.5 groups were respectively transferred to GF-zebrafish. In this study, DHA supplementation subsequently altered the effect of gut microbiota on lipid metabolism, as shown by boosting lipid oxidation independent of Cyclin D1, in zebrafish host. Although both nutrients and gut microbiota play important role in host metabolism, the composition and metabolism of the gut microbiota are mainly drived by diets (63). Thus, the effect gut microbiota in this study was the result of a secondary microbiota-host interaction drived by dietary DHA. The lipid-lowering effects mediated by Cyclin D1 was related to DHA entering hepatocytes, which was directly linked to the supplementation of DHA in diet. Thus the effect of DHA without involvement of gut microbiota is a primary effect. Moreover, the effect of DHA without involvement of gut microbiota modulated both lipid synthesis and β-oxidation, while the effect of gut microbiota modulated β-oxidation. This suggested a more comprehensive role of Cyclin D1 in modulating lipid metabolism. The effects of gut microbiota on host lipid metabolism might be mediated by its bacteria-derived components (64–66). Its dysbiosis can inhibit phosphorylation of AMPK thereby negatively influencing hepatic fatty oxidation and favoring lipogenesis resulting in excessive fat storage in the liver and obesity (67). However, the probiotic, *Lactobacillus*, and the microbial metabolite, butyrate, can activate AMPK and increase fatty acid oxidation (68). Further studies about the metabolites generated from DHA-altering gut microbial community might be beneficial to clarify the mechanism underlying the effects of gut microbita on lipid β-oxidation.

## Conclusion

Taken together, we described a regulatory mechanism in which DHA alleviates hepatic lipid synthesis and promotes lipid oxidation *via* the down-regulation of *Cyclin D1*. On the other hand, the gut microbiota formed by DHA-supplemented diet enhanced lipid oxidation *via* a mode independent on *Cyclin D1*. These results suggested when we use HFD in fish feeding, fatty acid composition in diet also should be taking into consideration to make sure containing appropriate DHA.

## Data Availability Statement

The datasets presented in this study can be found in online repositories. The names of the repository/repositories and accession number(s) can be found at: https://www.ncbi.nlm.nih.gov/, PRJNA771550.

## Ethics Statement

The animal study was reviewed and approved by Institute of Feed Research of Chinese Academy of Agricultural Sciences Animal Care Committee.

## Author Contributions

ZZho designed the research. QD wrote the paper and performed experiments and acquired data. ZZha gave conceptual advice for the paper and assisted in gut microbiota analysis. ER and REO reviewed and helped to revise the manuscript. QH and QZ participated in zebrafish husbandry and sampling. CR, YY, and ZZha co-analyzed and discussed the results. All authors read and approved the final manuscript.

## Funding

This work was supported by the National Natural Science Foundation of China (NSFC 31925038 and 32061133004) and the National Key R&D Program of China (2018YFD0900400).

## Conflict of Interest

The authors declare that the research was conducted in the absence of any commercial or financial relationships that could be construed as a potential conflict of interest.

## Publisher's Note

All claims expressed in this article are solely those of the authors and do not necessarily represent those of their affiliated organizations, or those of the publisher, the editors and the reviewers. Any product that may be evaluated in this article, or claim that may be made by its manufacturer, is not guaranteed or endorsed by the publisher.
