# Supplementary material for: DHA Suppresses Hepatic Lipid Accumulation via Cyclin D1 in Zebrafish
Source: Front Nutr. 2022 Jan 25;8:797510. doi: 10.3389/fnut.2021.797510 (PMC8823328; doi:10.3389/fnut.2021.797510)
Supplement: Supplementary file 1 [file Data_Sheet_1.pdf]

Supplemental Figure

Figure legend

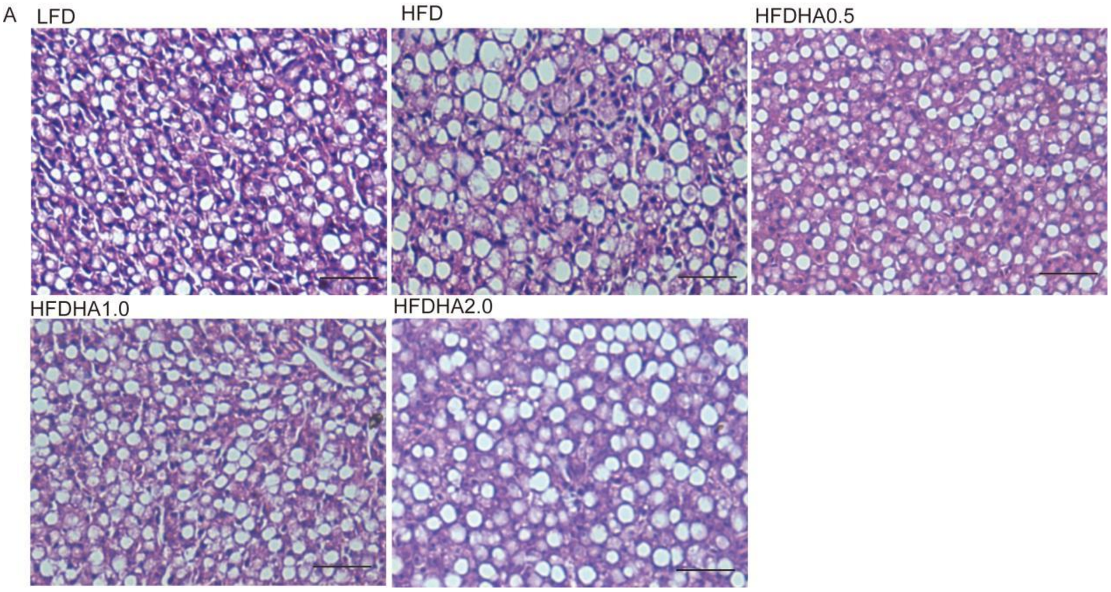

Supplemental Figure 1. H&E staining of liver sections of LFD, HFD and HFDHAs-fed zebrafish for 4 wks. The scale bar is 20 μm.

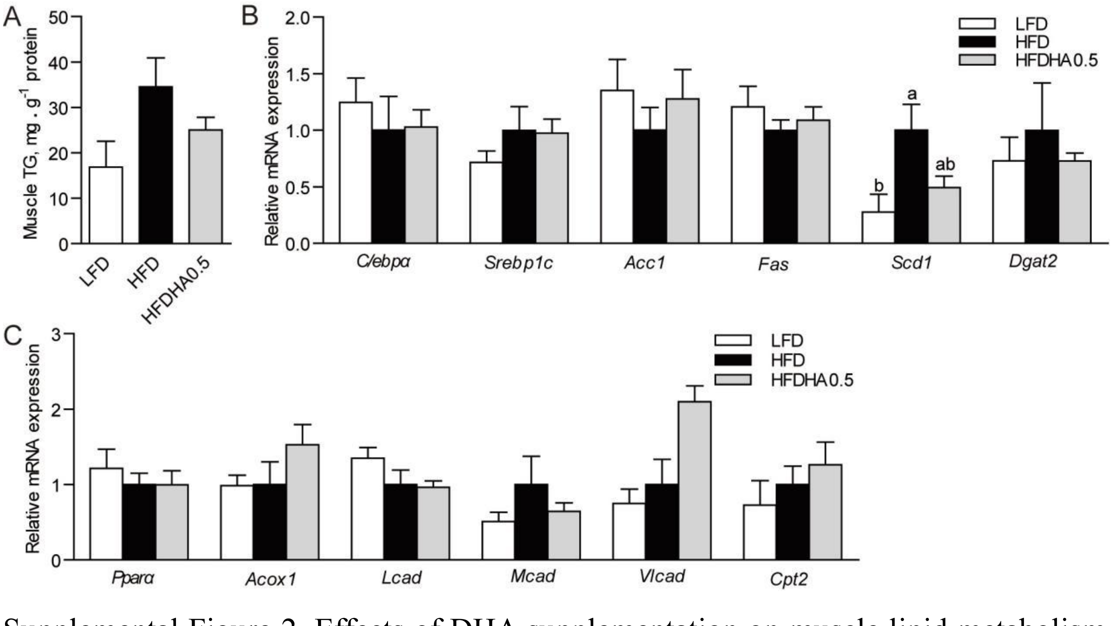

Supplemental Figure 2. Effects of DHA supplementation on muscle lipid metabolism. (A) Muscle TG content of zebrafish fed HFD and HFDHAs for 2 wks. Relative mRNA expression of genes related to (B) lipid synthesis and (C) β-oxidation. Values are means ± SEMs (n = 4 biological replicates). Means without a common letter are significantly different, $P < 0.05$.

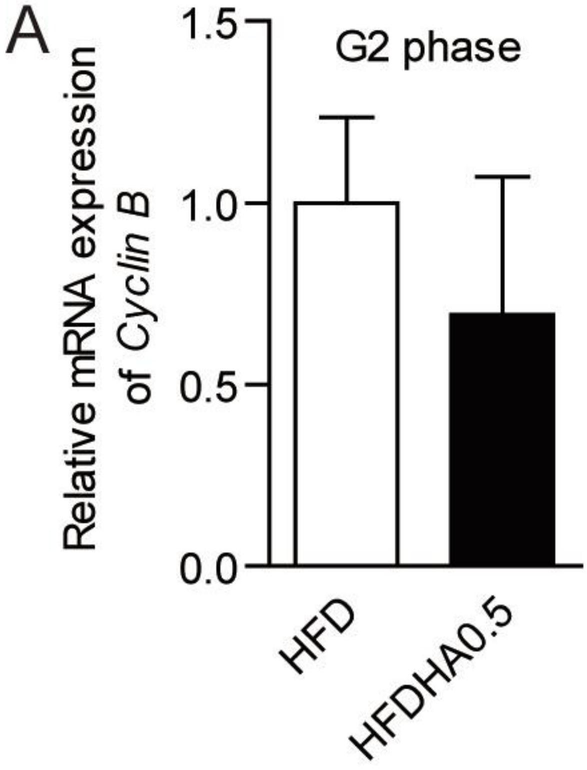

Supplemental Figure 3. Relative mRNA expression of genes encoding D-type Cyclins in the liver of LFD, HFD and HFDHA0.5-fed zebrafish. Values are means ± SEMs (n = 5 biological replicates).
